# Early results of full-endoscopic decompression of lumbar central canal stenosis by outside-in technique: A clinical and radiographic study

**DOI:** 10.1097/MD.0000000000027356

**Published:** 2021-10-01

**Authors:** Hyeun-Sung Kim, Sagar B. Sharma, Harshavardhan D. Raorane, Kyeong-Rae Kim, Il-Tae Jang

**Affiliations:** a Department of Neurosurgery, Nanoori Hospital Gangnam, 731, Eonju-ro, Gangnam-gu, Seoul, Republic of Korea; b Smt. S.C.L. General Hospital, Ahmedabad, India; c Nanoori Gangnam Hospital, 731, Eonju-ro, Gangnam-gu, Seoul, Republic of Korea ZIP-06048.

**Keywords:** degenerative spine, full-endoscopic decompression, laminectomy, lumbar canal stenosis, minimally invasive spine surgery, spinal endoscopy, stenosis

## Abstract

Retrospective cohort study.

Full-endoscopic decompression of lumbar spinal canal stenosis is being performed by endoscopic surgeons as an alternative to micro-lumbar decompression in the recent years. The outcomes of the procedure are reported by few authors only. The aim of this paper is to report the clinical and radiographic outcomes of full endoscopic lumbar decompression of central canal stenosis by outside-in technique at 1-year follow-up.

We reviewed patients operated for lumbar central canal stenosis by full endoscopic decompression from May 2018 to November 2018. We analyzed the visual analogue scale scores for back and leg pain and Oswestry disability index at pre-op, post-op, and 1-year follow-up. At the same periods, we also evaluated disc height, segmental lordosis, whole lumbar lordosis on standing X-rays and canal cross sectional area at the affected level and at the adjacent levels on magnetic resonance imaging and the facet length and facet cross-sectional area on computed tomography scans. The degree of stenosis was judged by Schizas grading and the outcome at final follow-up was evaluated by MacNab criteria.

We analyzed 32 patients with 43 levels (M:F = 14:18) with an average age of 63 (±11) years. The visual analogue scale back and leg improved from 5.4 (±1.3) and 7.8 (±2.3) to 1.6 (±0.5) and 1.4 (±1.2), respectively, and Oswestry disability index improved from 58.9 (±11.2) to 28 (±5.4) at 1-year follow-up. The average operative time per level was 50 (±16.2) minutes. The canal cross sectional area, on magnetic resonance imaging, improved from 85.78 mm^2^ (±28.45) to 150.5 mm^2^ (±38.66). The lumbar lordosis and segmental lordosis also improved significantly. The disc height was maintained in the postoperative period. All the radiographic improvements were maintained at 1-year follow-up. The MacNab criteria was excellent in 18 (56%), good in 11 (34%), and fair in 3 (9%) patients. None of the patients required conversion to open surgery or a revision surgery at follow-up. There was 1 patient with dural tear that was sealed with fibrin sealant patch endoscopically. There were 10 patients who had grade I stable listhesis preoperatively that did not progress at follow-up. No other complications like infection, hematoma formations etc. were observed in any patient.

Full endoscopic outside-in decompression method is a safe and effective option for lumbar central canal stenosis with advantages of minimal invasive technique.

## 1 Introduction

With increasing longevity, the size of geriatric population continues to rise. Degenerative conditions of the lumbar spine are common in this age group. Lumbar canal stenosis is the commonest cause for which surgery is performed in the geriatric population.^[1–3]^ At present, micro-lumbar decompression via interlaminar approach is the gold standard for this condition.^[4–6]^ Fusion is indicated in cases of instability and other indications. Although the iatrogenic muscle damage is less as compared to traditional open procedures, the technique of micro-lumbar decompression still causes injury to the posterior elements of spine.^[7–9]^ To overcome this problem, the technique of unilateral laminotomy and bilateral decompression using tubular retractors has been introduced. More recently, with constant development in field of optics and instruments, the technique of endoscopic decompression has also been reported.^[10–13]^ The development of larger size endoscopes, with wider working channels and larger instruments has made the surgery for spinal stenosis conceivable using endoscope. However, only few reports are available that show follow-up results of full endoscopic lumbar decompression technique.^[14–16]^ The aim of our paper was to analyze the clinical and radiological outcomes of lumbar central canal stenosis treated by full endoscopic decompression using the outside-in technique via the interlaminar approach.

## 2 Materials and methods

### 2.1 Patient population

The present study is approved by institutional review board of Nanoori hospital, Seoul, Republic of Korea. (NR-IRB 2019-008). The written consent was obtained from the patients participated in the study. We retrospectively analyzed the patients operated for lumbar central canal stenosis at our institute from May 2018 to November 2018 by full-endoscopic decompression. Inclusion criteria were patients with unilateral or bilateral radicular pain with magnetic resonance imaging (MRI) evidence of lumbar canal stenosis and failed conservative treatment. Exclusion criteria were patients with instability (defined as the translation of adjacent vertebrae by >11 mm or >4° on flexion-extension lateral X-rays), foraminal/extraforaminal stenosis and lack of 1-year follow-up MRI.

### 2.2 Operative technique

We perform the procedure under epidural or general anesthesia with the patient in prone position on a radiolucent table. We use a large diameter endoscope with integrated working channel having an outer diameter of 10 mm and working channel of 6 mm. A long diamond drill is used for bony work. A radiofrequency probe is used for tissue ablation and bleeding control. The initial landing is at the “V-point” of the interlaminar window that is the junction of the superior and inferior lamina with the facet joint. Using a diamond burr, we drill the lower half of the cranial lamina and the upper part of the caudal lamina until the edges of the ligamentum flavum is exposed. The medial part of the facet joint is drilled to decompress the lateral recess as well. After ipsilateral laminectomy, the base of the spinous process is drilled. The contralateral lamina is drilled in a similar manner by “over the top” technique. The ligamentum flavum is persevered till the bony drilling on both sides is complete. Lastly the flavum is removed and the decompression is completed using endoscopic kerrison punches. Since the epidural space is not entered before removal of the ligamentum flavum, we prefer to name the technique as an outside-in technique as compared to the traditional inside-out technique where the kerrison punches are introduced under the ligamentum flavum and removed piecemeal. (Video S1, Supplemental Digital Content: showing the illustrative animation of endoscopic stenosis lumbar decompression; Video S2, Supplemental Digital Content: illustrates an endoscopic view of the key surgical steps during full endoscopic lumbar canal stenosis decompression).

### 2.3 Demographic, clinical & radiographic data

**Figure 1:**
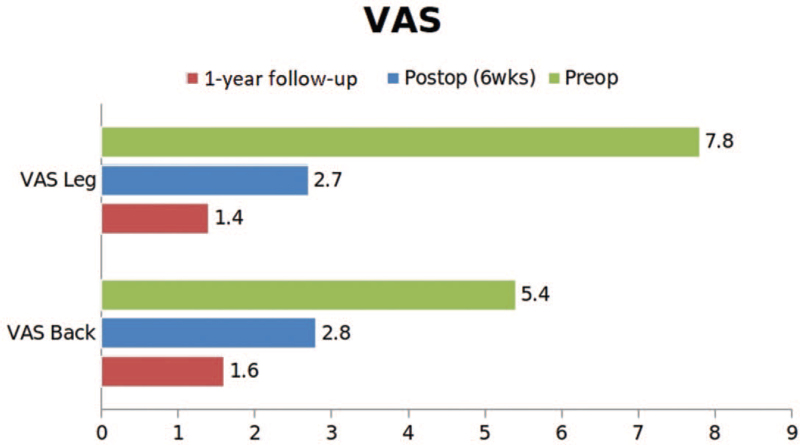
The VAS back and leg scores at preoperative, immediate postoperative, and 1-yr follow-up showing significant reduction in patients back and leg pain. VAS = visual analogue scale.

We analyzed demographic data including age, Body mass index, Charlson co-morbidity index, levels operated, blood loss & operative time. Clinical data included visual analogue scale (VAS) for back and leg pain and the Oswestry disability index (ODI). The final outcome was assessed using the Mac-Nab criteria at 1-year follow-up. The radiographic parameters included disc height, segmental lordosis angle, whole lumbar lordosis on standing X-rays; the length of the facet joint line and the cross-sectional area of the facet on both sides at the mid-discal level on computed tomography-scans; (see Fig. 1 demonstrates the method of measurement of facet length and cross-sectional area) and the canal cross-sectional area at the affected level and at levels above and below. The degree of listhesis, if present, was also evaluated. Instability was ruled out by dynamic X-rays. All these measurements were made preoperatively, postoperatively and at 1-year follow-up.

### 2.4 Statistical analysis

The quantitative variables were compared using *t* test and the qualitative variables were compared using chi-square test. A *P* value of <.05 was considered significant.

## 3 Results

### 3.1 Demographic & perioperative data

We analyzed 32 patients (with 43 levels) with an average age of 63 (±11) years. The most common level operated was L4-5. All the patients were followed up for at least 1-year with dynamic X-rays and MRI. Four patients had 9 associated disc herniation and 2 had associated synovial cysts which were addressed during surgery. The average operative time per level was 50 (±16.2) minutes. In all the patients, a unilateral approach and bilateral decompression were performed. All the patients had central canal stenosis (see Table 1, which illustrates the demographic data with complications of the procedure in current study).

**Table 1:** The demographic and perioperative variables.

Parameter	Variable
Age	63 (±11) yrs
Sex ratio (M:F)	14:18
Body mass index (BMI)	23.34 (±3.2)
Charlson co-morbidity index	2.4 (±1.7)
Levels involved	
L5S1	5
L4L5	26
L3L4	9
L2L3	1
L1L2	1
Operative time	50 (±16.2) min
Schizas grade	
• C	22
• D	20
No. of levels	
• 1-level	23
• 2-level	7
• 3-level	2
Complications	
Dural tear	1
Postoperative dysesthesia	6

### 3.2 Clinical and radiographic data

**Figure 2:**
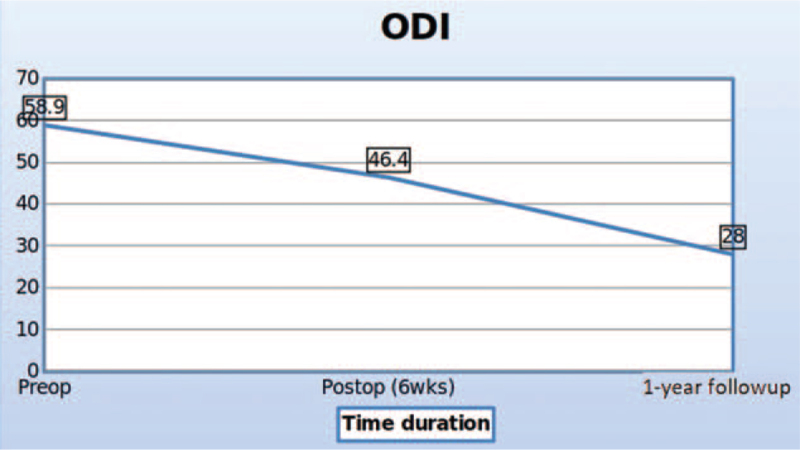
The ODI scores at preoperative, immediate postoperative, and 1-yr follow-up showing significant clinical improvement. ODI = Oswestry disability index.

**Figure 3:**
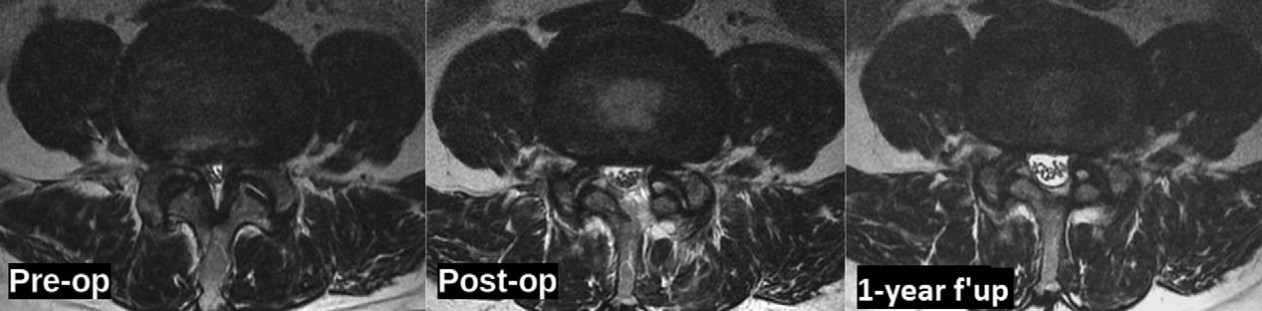
Case example of a 60yrs old female with L4-5 central canal stenosis with sacralized L5. The MRI scan axial cut done at preoperative, immediate postoperative, and 1-yr follow-up showing significant improvement in the lumbar canal cross sectional area at the operative level. MRI = magnetic resonance imaging.

**Figure 4:**
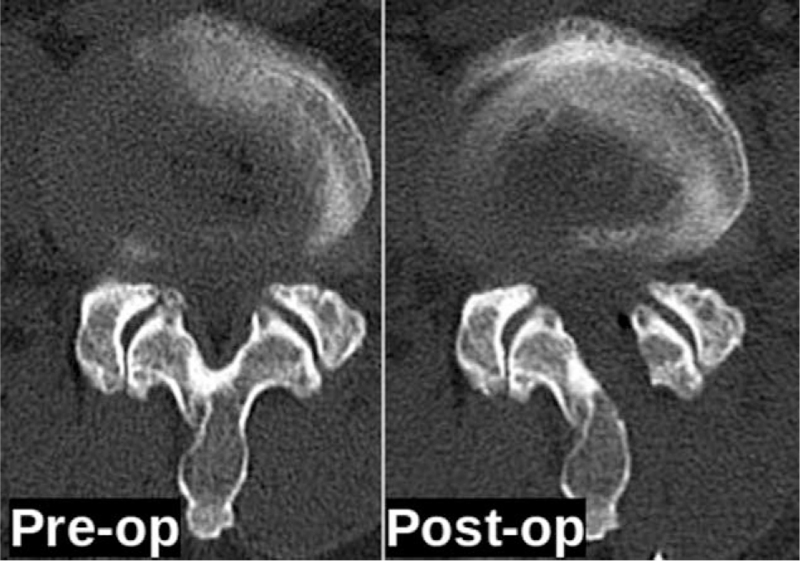
The CT scan axial cut of the same patient at preoperative and immediate postoperative point of time shows the approach from left side with preservation of ipsilateral and contralateral facets. CT = computed tomography.

**Figure 5:**
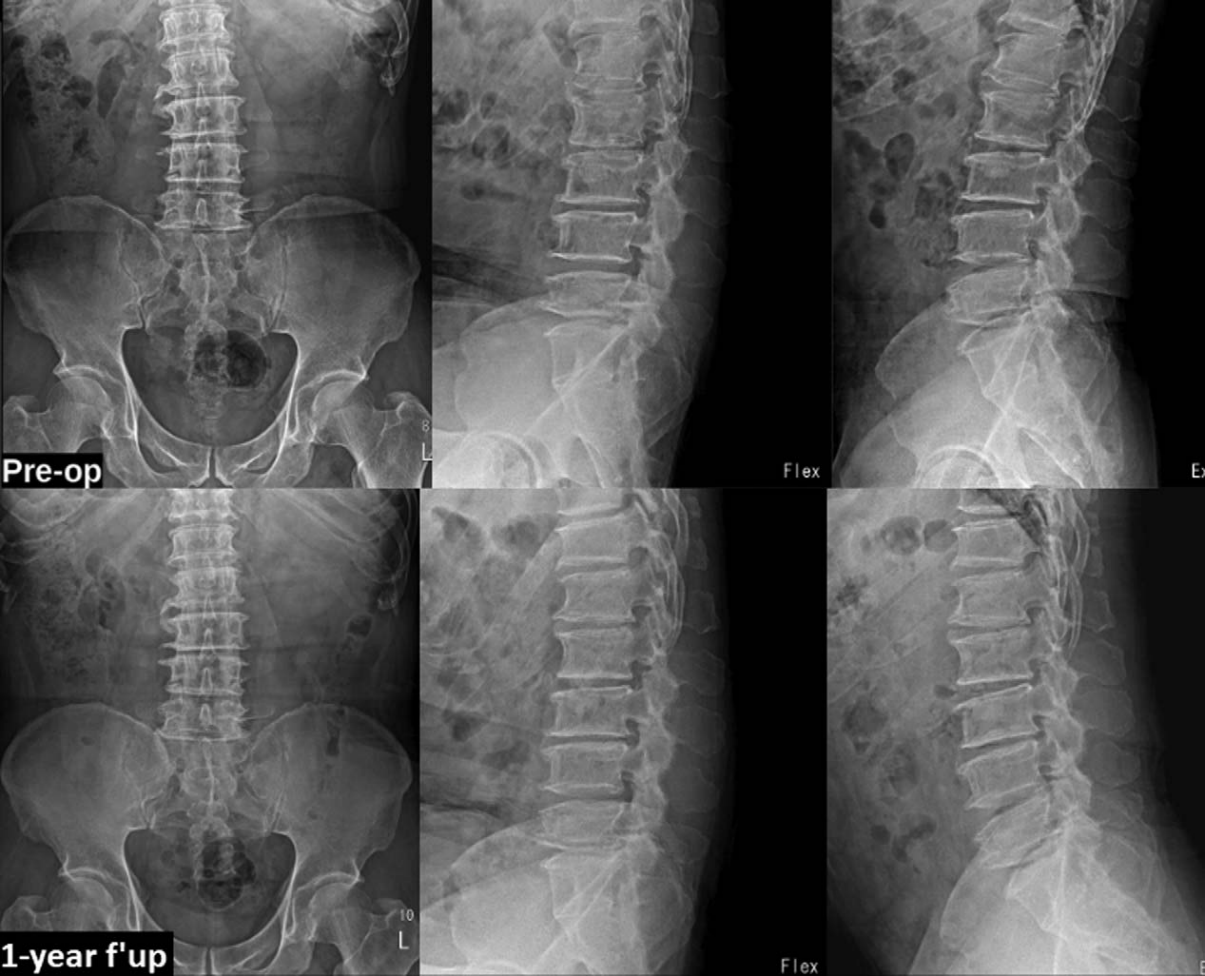
X-rays of the same patient comparing pre-operative and at 1-year follow-up shows no aggravation of instability.

**Figure 6:**
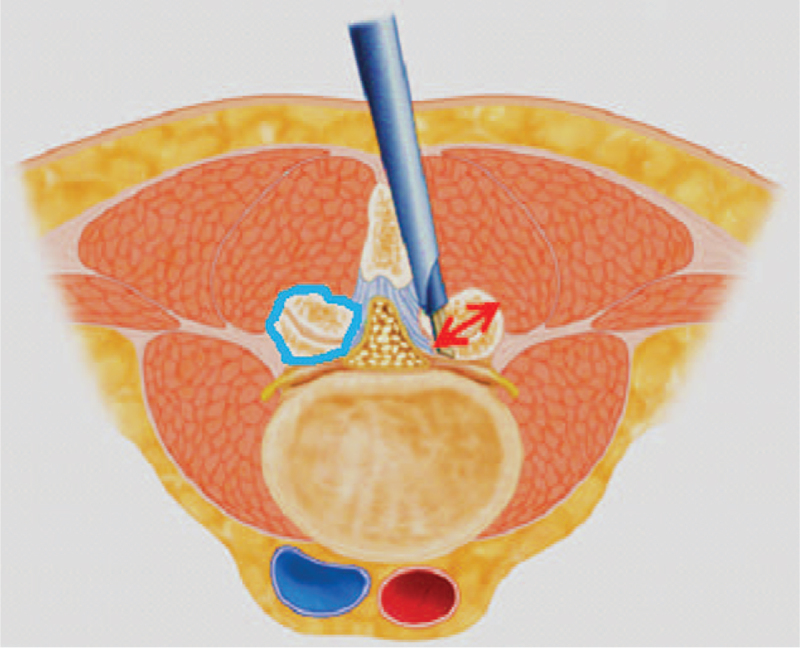
Diagrammatic representation of measurement of facet joint length and facet joint area. The measurements are taken at the mid-discal level on MRI/CT. The blue line on the left shows the measurement of the facet joint area, the red arrow on the right shows the measurement of facet joint length from postero-superior to antero-inferior.

The VAS back and leg improved from 5.4 (±1.3) and 7.8 (±2.3) to 1.6 (±0.5) and 1.4 (±1.2), respectively, (see Fig. 2, which illustrates significant improvement in VAS score of back and leg) and ODI improved from 58.9 (±11.2) to 28 (±5.4) at 1-year follow-up (see Fig. 3, which demonstrates significant improvement in ODI). The Mac-Nab criteria was excellent in 18 (56%), good in 11 (34%), and fair in 3 (9%) patients. The canal cross-sectional area on MRI improved from 85.78 mm^2^ (±28.45) to 150.5 mm^2^ (±38.66). None of the patients required conversion to open surgery or a revision surgery at follow-up (see Table 2, which illustrates the significant improvement in the spinal canal cross sectional area with maintenance of sagittal balance in long term follow-up). There was 1 patient with dural tear that was sealed with fibrin sealant patch endoscopically. There were 10 patients who had grade I stable listhesis preoperatively that did not progress at follow-up. No other complications like infection, hematoma formations etc were observed in any patient. All the patients reached the minimal clinically important difference of 3 points for VAS Back and Leg and 12 points for ODI at 6 weeks and 3 months follow-up, respectively. (Figs. 4, 5 and 6, respectively, demonstrates the preoperative and postoperative axial cut of MRI, computed tomography scan and dynamic radiographs of representative case).

**Table 2:** The radiographic results showing significant improvement in the lumbar canal cross sectional area at the operative level with preservation of ipsilateral and contralateral facets.

Variable	Preoperative	Postoperative	Final follow-up	*P* value
Disc height (mm)	10.6 (±2.89)	10.8 (±2.96)	10.56 (±3)	*P* > .05
Segmental lordotic angle	12.6 (±6.6)	15.26 (±7.78)	13.47 (±7.4)	*P* < .05
Lumbar lordosis	36.7 (±8.9)	41.6 (±10.16)	39.8 (±10.27)	*P* < .05
Facet length				
• Ipsilateral	14.8 (±0.9)	14.4 (±0.7)		*P* > .05
• Contralateral	14.9 (±0.6)	14.8 (±0.6)		*P* > .05
Facet cross-sectional area				
• Ipsilateral	240 (±12.83)	233 (±14.5)		*P* > .05
• Contralateral	241 (±15.4)	240 (±16.2)		*P* > .05
Canal cross sectional area				
• Index level	85.7 (±28.4)	151.3 (±38.6)	150.5 (±38.6)	*P* < .05
• Level above	130.8 (±21.9)	130.7 (±23.7)	130.4 (±26.6)	*P* > .05
• Level below	139.1 (±28.1)	139.8 (±28)	141.7 (±25.3)	*P* > .05

## 4 Discussion

Surgery for lumbar spinal stenosis dates back to 1930s when Mixter and Barr reported the first laminectomy for removal of herniated disc.^[17]^ Prior to this too, the procedure of laminectomy had been reported for infections, tumor, and trauma with unpromising results by few authors in the late 1980s.^[18,19]^ With further experience, the procedure gained acceptance among surgeons. McColluch was one of the pioneers of modern-day lumbar microsurgery who encouraged the use of microscope for lumbar procedures.^[20,21]^ In the 90s, Foley and Smith introduced the tubular retractor system of back muscle preservation.^[22]^ In the late 90s, the use of endoscope for lumbar disc herniations gained acceptance.^[23]^ With further development in technology, larger endoscopes were introduced which began to be used for lumbar decompression procedure via interlaminar approach. Numerous reports describing the use of endoscope for lumbar decompression procedures have been published.^[12–15,24–26]^ However, only few reports focus on the follow-up results of the procedure. Our study is the first to report the radiographic outcomes at 1-year follow-up. In our series, there was statistically significant improvement in the VAS back and leg scores at 6 weeks follow-up. The improvement in the ODI score was also significant, but improved later than the VAS score at 3-month follow-up. This signifies that although the radicular pain subsides immediately after surgery, the improvement in the disability takes time. The minimum clinically important difference for VAS of 3 points and ODI of 15 points was achieved in all the patients.^[27,28]^ The canal cross-sectional area improved significantly as well. The improvement was maintained at follow-up. We also evaluated the canal cross-sectional area at 1 level above the below the index level to see if there was a deterioration at the adjacent levels as a result of the surgery. This evaluation yielded insignificant differences indicating that endoscopic decompression does not accelerate adjacent segment degeneration. The disc height was also maintained. Evaluation of the facet length and facet area on the approach side and the contralateral side was also done (Fig. 1). There was insignificant reduction in both the parameters on both sides. This shows that facet injury is minimal with endoscopic decompression.^[11,13]^ The lumbar lordosis and the segmental lordosis also improved significantly. This is probably because once the compression of the canal is relieved; the compensatory obliteration of lumbar lordosis dissipates.^[29,30]^ The improvement in all these radiographic indices was maintained at follow-up indicating the medium-term efficacy of the technique. The complication rate was low. This is probably because the operating surgeon had significant experience with endoscopic spine procedures. None of the patients required conversion to open surgery or a revision surgery at follow-up. One patient had dural tear that was identified intraoperative & was sealed with fibrin sealant patch endoscopically. There were 10 patients who had grade I stable listhesis preoperatively that did not progress at follow-up. No other complications such as infection, hematoma formations etc, were observed in any patient. Komp et al^[31]^ described their mid-term results of full endoscopic decompression in lumbar spinal stenosis patients in few papers. They also compared full endoscopic decompression with micro-lumbar decompression and found favorable results.^[14,15]^ A recent study compared uniportal and biportal endoscopy with micro-lumbar decompression and found similar clinical outcomes.^[25]^ The authors of this study concluded that endoscopic procedures had the advantage of minimal postoperative pain. This is probably because of minimal muscle damage during endoscopic procedures. The outside-in technique is probably safer because the dura is shielded by the ligamentum flavum until the bony work is completed with the drill. The endoscope allows enhanced visualization and magnification that probably also increases the safety of the procedure. The patient can be mobilized the very next day after the procedure. Deterioration in the surgical results has been described in literature with conventional procedures due to resection of the spinous process and the interspinous ligaments.^[32–34]^ However, with endoscopic procedures, the iatrogenic resection of these elements is avoided that seems capable of reducing the operation induced consequences. Thus, the ideal goal of surgery for lumbar spinal stenosis of sufficient decompression with minimal operation induced damage seems plausible with endoscopic decompression procedures. However, the study is not without limitations. These include the selection bias and other disadvantages of retrospective study. The sample size is small and the follow-up period is rather short. This is a single center, single surgeon study. Future studies with larger sample size, a longer follow-up, and a prospective design are warranted.

## 5 Conclusion

Full-endoscopic decompression of lumbar central canal stenosis may be considered as a viable alternative to micro-lumbar decompression with added advantage of being the most minimally invasive and advanced technique of spinal decompression.

## Author contributions

**Conceptualization:** Hyeun-Sung Kim, Il-Tae Jang.

**Data curation:** Sagar B. Sharma, Kyeong-Rae Kim.

**Methodology:** Sagar B. Sharma, Harshavardhan D. Raorane, Kyeong-Rae Kim, Il-Tae Jang.

**Supervision:** Hyeun-Sung Kim, Il-Tae Jang.

**Validation:** Hyeun-Sung Kim, Il-Tae Jang.

**Writing – original draft:** Sagar B. Sharma, Harshavardhan D. Raorane.

**Writing – review & editing:** Hyeun-Sung Kim, Sagar B. Sharma, Il-Tae Jang.

## Supplementary Material

Supplemental Digital Content

## Supplementary Material

Supplemental Digital Content
